# Investigating the Suitability of Mare’s Milk-Derived Exosomes as Potential Drug Carriers

**DOI:** 10.3390/biom14101247

**Published:** 2024-10-01

**Authors:** Shynggys Sergazy, Sanzhar Zhetkenev, Zarina Shulgau, Laura Chulenbayeva, Yevgeniy Kamyshanskiy, Madiyar Nurgaziyev, Ayaulym Nurgozhina, Zhanel Mukhanbetzhanova, Kulzhan Berikkhanova, Alexander Gulyayev, Mohamad Aljofan

**Affiliations:** 1 National Laboratory Astana, Private Institution, Nazarbayev University, Astana 010000, Kazakhstanlaura.chulenbayeva@nu.edu.kz (L.C.); madiyar.nurgaziyev@nu.edu.kz (M.N.); zhanel.pernebek@nu.edu.kz (Z.M.); akin@mail.ru (A.G.); mohamad.aljofan@nu.edu.kz (M.A.); 2 Laboratory and Pathological Diagnostic Unit, Karaganda State Medical University, Karaganda 100000, Kazakhstan; 3 Department of Biomedical Science, Nazarbayev University School of Medicine, Astana 020000, Kazakhstan

**Keywords:** exosomes, extracellular vesicles, quercetin, aging, ROS

## Abstract

Exosomes are cell-derived, membrane-surrounded particles that deliver bioactive molecules to various cells. Due to their small size, low immunogenicity, extended blood circulation, and involvement in cellular communication, they hold potential as effective drug carriers. Exosomes are present in various biological fluids, including mare’s milk, a traditional drink in Central Asia. This study aims to compare exosome isolation methodologies and determine the stability of mare’s milk-derived exosomes as potential therapeutic carriers. Three extraction methods—immunoprecipitation, size exclusion chromatography, and total exosome isolation—were compared in terms of exosome characteristics, purity, and content. The isolated exosomes were then loaded with quercetin, and their ability to increase its bioavailability was tested in vitro and in vivo. Total exosome isolation was identified as the most efficient method for producing high-quality exosomes. These exosomes were loaded with quercetin and compared to free quercetin and exosomes alone. Exosomes loaded with 80 µM quercetin significantly restored β-galactosidase activity and cellular viability in doxorubicin-treated cells, exhibiting similar potency to 160 µM free quercetin. In aged model animals, treatment with quercetin-loaded exosomes resulted in significantly less acute and subacute damage to the myocardium, kidneys, and liver compared to untreated control animals. This study provides a proof-of-concept that mare’s milk-derived exosomes can be effectively absorbed by cells and animal tissues, supporting their potential use as drug carriers.

## 1. Introduction

Extracellular vesicles (EVs) including exosomes, microvesicles, and apoptotic vesicles, are membrane-bound small structures released from cells into the surrounding environment [1]. They play essential roles in intercellular communication, particularly exosomes, which contain several constituents from the cells that secrete them and appear to be involved in the pathogenesis of various disorders, including cancer, neurodegeneration, and inflammatory diseases [2]. Interestingly, the biocompatibility of exosomes, their circulating stability, and bioavailability in vivo, have allowed them to gain increasing attention as an emerging drug delivery methodology over the last decade [3]. Due to these unique properties, exosomes have gained significant attention as potential therapeutic agents and drug delivery systems [4,5] Their natural origin and ability to traverse biological barriers make exosomes ideal candidates for delivering bioactive compounds in a variety of biomedical applications [6,7]. They represent an important pathway to transfer information between cells, and, thus, might be developed to package and deliver therapeutic molecules. Although exosomes are not nanoparticles derived from the nanotechnology due to its non-human nature, they may act as nano-carriers owing to their particle diameter [8], which is estimated to be between 30–100 nm, and, thus, can be used to load a variety of small bioactive molecules to improve bioavailability. Therefore, exosomes’ particle sizes allow them to penetrate deep into the tissues and overcome barriers, such as the blood–brain barrier and the deformable cytoskeleton. Another important characteristic is that they have a slightly negative zeta potential, which guarantees their long-term circulation [9]. In addition, some exosomes are capable of escaping from the immune system and have been shown to have a low immunogenicity and high stability in the blood, which prolongs drug circulation within the body [10]. Recent studies have particularly focused on the therapeutic potential of milk-derived exosomes. Bovine milk exosomes, in particular, have been explored for their ability to carry proteins, miRNA, and other bioactive molecules that can enhance tissue regeneration, reduce inflammation, and support immune modulation [5]. However, little attention has been paid to mare’s milk exosomes, which may offer unique benefits due to differences in composition and bioactive content [11]. Exosomes isolated from milk are biocompatible and present an ideal platform for delivering compounds, such as quercetin.

Mare’s milk is the national drink of the indigenous population in Central Asia, including Kazakhstan. Recent scientific data on the characteristics of the composition of horse milk and their potential properties that contribute to improving health have increased interest in this dietary source [12]. Mare’s milk shares some similarities to human breast milk and, therefore, may have some valuable therapeutic properties [13]. Mare’s milk has a very good hygienic and sanitary status and differs from the milk of other farm animals in that it has the lowest somatic cell content and a very low total number of microorganisms [14]. There are no studies examining the use of mare’s milk-derived exosomes as a form of therapeutic carrier. Therefore, the current study aims to extract mare’s milk-derived EVs and isolate high content exosomes to test their suitability as a reliable form of therapeutic drug carrier. However, milk-derived EVs significantly differ in size, ranging from 30 nm–10 µm, and possibly contain various constituents; thus, we do not exactly know which EV extraction methods would allow us to have the optimum exosomal size. Thus, there is a need to compare and characterize different methods that allow the isolation of the highest concentration of exosomes with the most homogenous shapes and sizes suitable for use as therapeutic drug carriers. Hence, we will use quercetin, a naturally occurring flavonoid, as the drug to confirm the suitability. Interestingly, quercetin has shown a significant effect in mitigating oxidative stress, inflammation, and senescence, which are central to age-related tissue damage [15]. However, despite its potential therapeutic effect, quercetin has a significantly poor bioavailability and rapidly degrades in the gastrointestinal tract [16]. Hence, using mare’s milk-derived exosomes to encapsulate quercetin would enable us to investigate both the effectiveness of exosomes as a drug carrier and quercetin as an antiaging molecule.

## 2. Materials and Methods

### 2.1. Mare’s Milk Purchase

Fresh mare’s milk was purchased from Saumal factory located in the Karaganda region, Kazakhstan, then aliquoted in 50 mL tubes and stored at −80 °C. To remove milk fat globules and cell debris, thawed samples were centrifuged at 10,000× *g* for 30 min at 4 °C. To obtain a large number of good quality exosomes, three exosome isolation methods were used.

### 2.2. Exosomes Isolation Methods

#### 2.2.1. Total Exosome Isolation

The first method used was total exosome isolation (TEI), where milk samples were centrifuged at 2000× *g* for 30 min, and then the supernatant was collected and 2.5 mL of total exosome isolation reagent (Invitrogen by life technologies, Waltham, MA, USA) was added. The work was carried out according to the manufacturer’s guidelines [Thermo Fisher Scientific, Waltham, MA, USA], where TEI reagents were added to mare’s milk (1:2 ratio), mixed well until homogenous, and then incubated overnight at 4 °C. The solution was then centrifuged at 10,000× *g* for 1 h at 4 °C. The supernatant was discarded; the pellet and sediment exosomes were resuspended in an equal volume of 1 × PBS.

#### 2.2.2. Isoelectric Precipitation

Isoelectric precipitation (IP), was used as a second method to extract exosomes. Briefly, skimmed milk was diluted with distilled water, and the pH was adjusted to 4.6. Samples were then centrifuged at 5100× *g* for 20 min at 25 °C, and the supernatant was collected and filtered using 0.45 µm filters.

#### 2.2.3. Size Exclusion Chromatography

The third isolation method was size exclusion chromatography (SEC), and this was performed according to the manufacturer’s instructions [IZON Science, Christchurch, New Zealand, qEV columns]. This involved isolating exosomes from the supernatant of cell cultures and complex biological fluids. Since the separation is based on size, the vesicles pass through the column, are retained, and are then eluted in the void. Proteins and other contaminants that are smaller than the pores are retained by the column and eluted later. Isolation using original size exclusion columns removes >99% of background protein contaminants and up to 95% of high-density lipoprotein contaminants from samples in a single isolation. Column qEV original 70 (IZON Science, Christchurch, New Zealand), which has an optimal recovery range from 70 nm to 1000 nm, was used in the experiment. Columns were washed using filtered 1 × PBS, followed by a second wash containing 19 mL of filtered 1 × PBS and 0.5 mL of supernatant. A total of 5–10 concentrated fractions were collected for the study.

### 2.3. Characterization of Exosomes

#### 2.3.1. Transmission Electron Microscopy

Approximately 5 µL of exosomes, isolated using SEC, IP, or TEI, were added onto a carbon film-supported copper grid (Sigma-Aldrich, St. Louis, MO, USA) for transmission electron microscopy (TEM) analysis. The concentrated samples were maintained in a sterile environment and kept still in order to allow the liquid to evaporate. Samples then were fixated and characterized using a JEM-1400 Plus electron microscope (JEOL Ltd., Akishima, Japan).

#### 2.3.2. Nanoparticle Determination

Exosome particle size determination was performed using a Malvern Zetasizer Nano-ZS ZEN 3600 (Malvern Panalytical, Malvern, UK). Approximately 350 µL of obtained exosomes were used in the experiment, and these were measured at 25 °C ambient temperature.

#### 2.3.3. Protein Determination

The Thermo Scientific™ Pierce™ BCA Protein Assay Kit was used to determine the protein concentrations in each of the exosome samples obtained using the different isolation methods, namely IP, TEI, and SEC. Bovine serum albumin (BSA) in 1% SDS was used as a standard, with final concentrations of 2 µM, 1 µM, 0.5 µM, 0.25 µM. and 0.125 µM. The BCA reagent (50 mL of solution A and 1 mL of solution B) was added into triplicates of exosome-containing media from the different isolation methods. A series of standards and 10 µL of each sample were added into a 96-well plate, followed by 190 µL of 1% SDS and 200 µL of BCA reagent. The plate was incubated for 30 min, and then read at a wavelength of 562 nm. A BCA total protein standard curve was obtained, and the protein concentrations were calculated. The Kruskal–Wallis test was performed to assess whether there were statistically significant differences in the medians of protein content in each of the media obtained by different methods.

#### 2.3.4. Western Blot Analysis

All of the following antibodies were purchased from Thermo Fisher Scientific, Waltham, MA, USA: MFGE8 (lactadherin) monoclonal antibody (EDM45), MFGE8 (lactadherin) polyclonal antibody, and CD63 goat polyclonal antibody. These antibodies were used for Western blot analyses. Samples containing EV pellets were suspended in PBS and diluted with lysis buffer, then further mixed with 6x SDS sample buffer and placed at 95 °C for 5 min. Gel (12% Mini-PROTEAN TGX Stain-Free, Bio-Rad, Hercules, CA, USA) electrophoresis was run for 20–25 min at 90 V for stacking, and then at 1–1.5 h at 110 V for separating. The proteins were transferred using a Transfer-Blot Turbo Transfer System with a transfer pack (Bio-Rad). The membranes were blocked with 5% non-fat dried milk dissolved in TBST. The proteins were detected by incubation with a secondary antibody conjugated with HRP (anti-rabbit IgG goat antibody, anti-rabbit IgG donkey antibody, and anti-goat IgG donkey antibody, all from Invitrogen, Waltham, MA, USA). The membranes were washed thrice prior to imaging. We detected the peroxidase activity using a Clarity^TM^ Western ECL substrate (Bio-Rad) and visualized it using an ChemiDoc^TM^ Imaging system (Bio-Rad).

#### 2.3.5. Exosome Loading with Quercetin

The quercetin loading was performed according to [12]. Approximately, 99 mL of exosomes extracted from the mare’s milk was mixed with 14 mL of quercetin dissolved in 1% DMSO solution to a achieve a final concentration of 160 μM. The solution was left to mix with magnet stirrer for 24 h. In order to remove the unincorporated quercetin and to purify the solution, IZON ASF and qEV original 70 (IZON Science, Christchurch, New Zealand) columns were used.

### 2.4. In Vitro Assays

#### 2.4.1. Cell Cultures

Neonatal human dermal fibroblast (HDFn) cells were cultured in DMEM high-glucose medium with L-glutamine and with sodium pyruvate (Biowest, Nuaillé, France), supplemented with 10% exosome-depleted fetal bovine serum (Gibco; Thermo Fisher Scientific, Inc.) and 1% penicillin–streptomycin solution (Sigma) in a 37 °C, 5% CO_2_ incubator (Binder CB-150, Binder, Tuttlingen, Germany). Aging was induced by following the protocol described by Bjientinesj and colleagues [17], where cells with ~80% confluency were treated with a final concentration of 3μM of doxorubicin (DOX) for 3 h, and then washed with HBSS. To determine the effect of exosomes, cells were incubated with exosomes alone from different isolation methods (IP, SEC, or TEI) and then loaded with quercetin and compared to quercetin only using different concentrations (20, 40, 80, and 160 μM). For the vehicle control, cells were treated with 1% DMSO, and for the negative control, cells were treated with 1 × PBS.

#### 2.4.2. Cell Viability

To determine cell viability, an MTT assay kit (10009365, Cayman, Ann Arbor, MI, USA) was used, which is a colorimetric assay that measures cellular metabolic activity as an indicator of cell viability, proliferation, and cytotoxicity [Merck]. Briefly, cells were seeded in 96-well plate (10^5^ cells/well) in 200 μL of DMEM medium for 24 h. Untreated cells were used as a negative control. After incubation, 10 μL of MTT reagent was added to each well, gently mixed for 1 min on orbital shaker, and then incubated for 3 h at 37 °C in 5% CO_2_. Furthermore, 100 μL of crystal dissolving solution was added to each well to dissolve the formazan crystals, producing a purple solution, which was incubated for 18 h at 37 °C in a CO_2_ incubator. The absorbance of each sample were measured at 570 nm on a BioTek Citation 5 Cell Imaging Multimode reader.

#### 2.4.3. β-Galactosidase Assay

β-galactosidase assay was performed using Mammalian β-Galactosidase Assay Kit (Thermo Fisher Scientific, Inc.), which is another colorimetric assay that measures metabolic activity as an indication of cell senescence [18]. Cells were plated in 96-well plate for 24 h, after that washed with 100 μL PBS (pH 7.2). A 100 μL of β-Galactosidase Assay Reagent was added to each well and incubated for 30 min at 37 °C. The reaction was stopped by adding 100 μL of β-Galactosidase Assay Stop Solution. Absorbance of cells were measured at 405 nm on BioTek Citation 5 Cell Imaging Multimode reader.

### 2.5. Animal Experimentations

#### 2.5.1. Efficacy of Quercetin-Loaded Exosomes from Mare’s Milk Compared to Free Quercetin in an Aging Male Rat Model

The efficiency of quercetin-loaded exosomes derived from mare’s milk versus free quercetin were evaluated using an aging model of male Wistar outbred rats. Experimental groups consisted of males older than 12 months, with young male Wistar rats aged 8 weeks serving as the negative control. On average, the rats weighted 200  ±  20 g and were housed in the animal facility of the National Center for Biotechnology, Astana, Kazakhstan. After a one-week adaptation period, the rats were randomly divided into their groups (3 rats/cage) and housed in a room with a controlled temperature and a 12 h light–dark cycle with unlimited access to standard food and drinking water ad libitum. 

#### 2.5.2. Experimental Design

The experiment was conducted on total of fifteen male rats (12 months old as aging sample, n = 12), plus young rats as a control (n = 3), divided into five groups of three rats per group as follows: Group 1 (exosomes-only group) contained old rats that were administered intragastrically with empty exosome from mare’s milk once daily for a week. Group 2 (quercetin-loaded exosomes) contained old rats administered intragastrically with quercetin-loaded exosomes from mare’s milk (quercetin concentration 2 mg/mL, volume administered was 0.5 mL per rat, equating to 1 mg of quercetin per rat) once daily for a week. Group 3 (quercetin only) had old rats administered intragastrically with free quercetin (quercetin concentration 2 mg/mL, volume administered was 0.5 mL per rat, equating to 1 mg of quercetin per rat) once daily for a week. Group 4 (control, untreated old rats) contained old rats that were administered intragastrically with drinking water (manipulation control) at a volume of 1 mL per rat. The fifth group (control. untreated young rats) had young rats that were administered intragastrically with drinking water once daily for a week. On the conclusion of the experiment (day 8), animals were killed and three major organs were harvested for analysis, namely the heart, kidneys, and liver. Organs were preserved by immersion in 10% neutral buffered formalin.

#### 2.5.3. Histopathological Examination

Collected tissues were fixed and sectioned using a standard protocol developed by the Veterinary Diagnostic Laboratory at Washington University [19], ensuring each animal was examined for the same organ slice. After sectioning, the samples underwent processing with isopropyl alcohol, xylene, and paraffin using a tissue processor. Once placed in the histo-cassettes, the material was embedded in paraffin forming blocks for histological sectioning. Histological sections of approximately 3 μm were stained with hematoxylin and eosin to determine the general morphological pattern and cellular infiltration in the heart, kidneys, and liver. Also, Masson’s trichrome staining was used to identify collagen fibers, which were stained blue, and connective tissue in the heart and liver. To detect mucopolysaccharide accumulation in the mesangium and basal membranes of the glomeruli and tubules of the kidneys and to assess glycogen accumulation in hepatocytes, tissue were stained with periodic acid–Schiff (PAS). Sections were examined using light microscopy using a Zeiss AxioLab 4.0 microscope at magnifications of ×40, ×100, ×200, and ×400. AxioVision 7.2 software for Windows was used for image capturing.

#### 2.5.4. Morphometric Study

The morphometric study was conducted by two independent histopathologists experienced in animal models. They were not aware of which animal group the images came from and analyzed each criterion separately. Lesions identified in each organ were described according to the International Harmonization of Nomenclature and Diagnostic Criteria (INHAND) [20,21].

A semiquantitative scoring analysis of the electron microscope images was used to evaluate the degree of ultrastructural damage, as previously described by Sergazy et al., 2020, which utilizes a four-point scale (Grade 1–4) for the morphological assessment of subcellular structures [22,23]. In brief, slides assessed for different morphologic changes, and lesions were classified as present/absent on a scale from 0 to 1 or assessed for severity on a scale from 0 to 4. Histopathological lesions of the heart were evaluated as present (“1”) or absent (“0”) included cardiomyocyte hypertrophy, focal hyper-eosinophilia, enlargement of cardiomyocyte nuclei/multinucleated cardiomyocytes, cellular infiltrate, and atherosclerosis. Fibrosis (perivascular and interstitial) was assessed on the following scale: “0”—none, “1”—minimal changes, “2”—mild changes, “3”—moderate changes, and “4”—severe lesions. Kidney lesions were evaluated as present (“1”) or absent (“0”), and included tubular epitheliocyte vacuolization and hyperplasia, formation of protein cylinders in tubules, interstitial cellular infiltrate, thickening and fibrosis of glomerular membranes, hyaline glomerulopathy, and glomerulosclerosis. Liver lesions were evaluated as present (“1”) or absent (“0”), and included focal hepatocyte vacuolization, presence of hyperchromatic/double-nucleated hepatocytes, lymphocytic infiltration, hyperplasia (cysts) of bile ducts, and periportal fibrosis. Glycogen accumulation was differentiated on a scale from 0 to 3, as follows: “0”—less than 10% of hepatocytes, “1”—11–30% of hepatocytes, “2”—31–60%, and “3”—more than 61% of hepatocytes.

### 2.6. Statistical Analysis

All data are expressed as mean  ±  standard error of the mean (S.E.M.) unless stated otherwise. Data were analyzed using one-way analysis of variance (ANOVA) and Student *t*-tests. Results with *p*-values less than 0.05 (*p*  <  0.05) were considered statistically significant. All experiments were performed in triplicate, unless stated.

## 3. Results

### 3.1. Comparison of Exosome Isolation Methods

**Figure 1:**
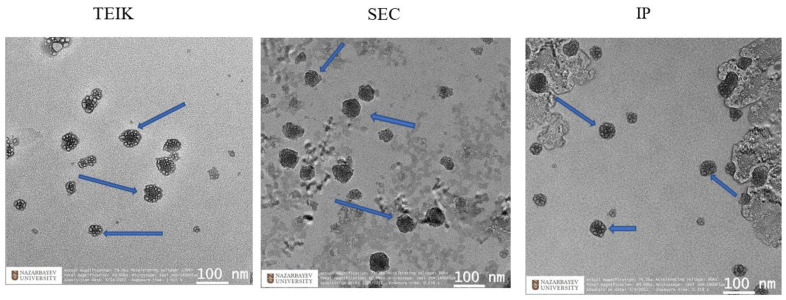
Morphology of exosomes obtained by the SEC, IP, and TEIK methods visualized by transmission electron microscopy. Arrows indicate exosomes.

A comparison of the three different methods of exosome isolation was performed to evaluate the morphology, properties of the obtained exosomes, and their applicability as drug carriers. The morphology assessment of the isolated exosomes performed using TEM shows that exosomes isolated using the different methods have morphologically dense vesicular structures, as indicated with arrows (Figure 1). It can be clearly observed that some debris can be observed in EVs obtained by IP and SEC, but not in the EVs isolated using the TEI method, which might be due to the PBS dilution during the isolation process.

**Figure 2:**
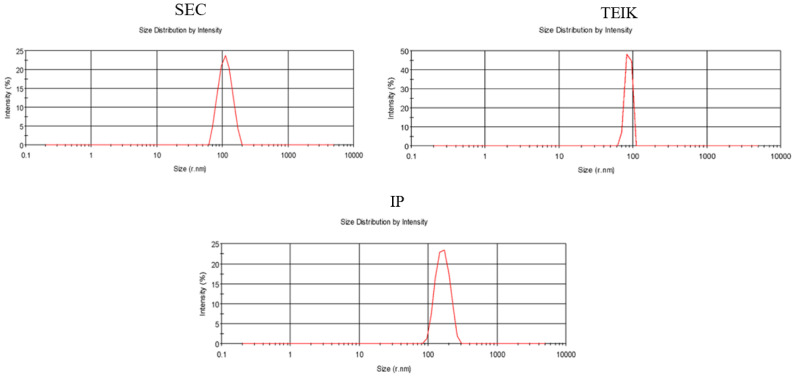
Nanoparticle detection illustrates the distribution of particles across the different isolation methods. Red line demonstrates size distribution by Intensity.

Nanoparticle tracking illustrating the size distribution of the particles shows the distribution of particles close to 100 nm (Figure 2). Particles isolated by SEC showed less variation in particle distribution than IP and TEI. There was no significant difference in the mean size of exosomes across the different isolation methods, namely SEC, TEIK, and IP (110 ± 8 nm, 118 ± 13 nm, and 131.5 ± 16 nm, respectively).

**Figure 3:**
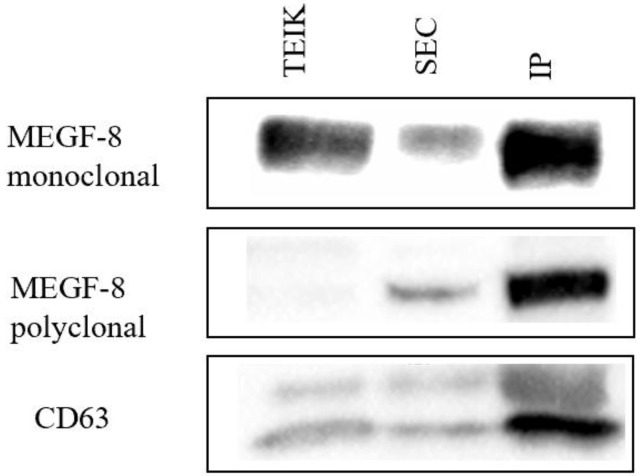
Western blot analysis using MEGF-8 monoclonal, MEGF-8 polyclonal, and CD63 antibodies with different EV isolation methods. Original images can be found in Figure S1.

**Figure 4:**
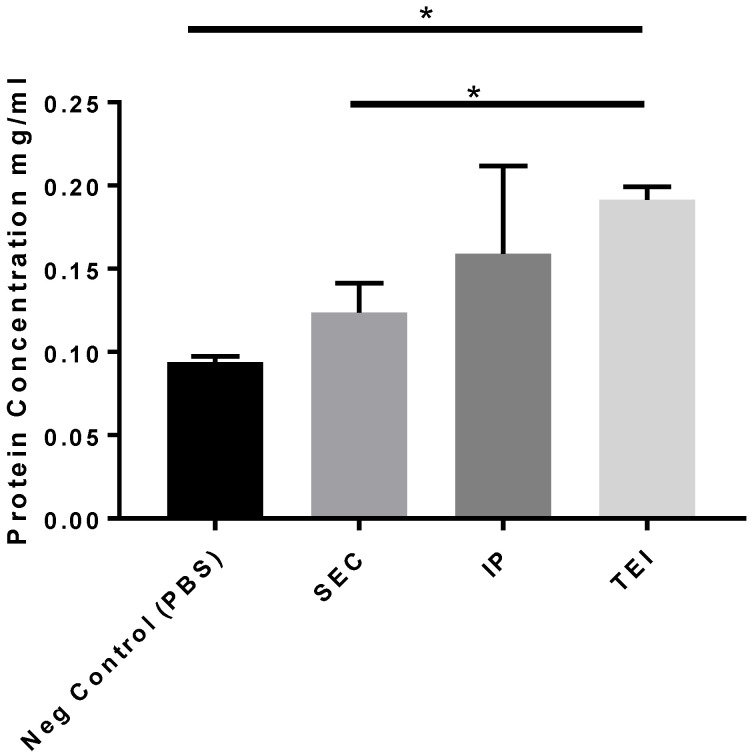
BCA protein determination analysis across methods with PBS control. SEC = size exclusion chromatography; IP = immunoprecipitation; TEI = total exosome isolation (TEI). All determinations were performed in triplicate; the error bars are the standard deviation. * designates significance at *p* < 0.05.

Western blot analysis using membrane markers, namely MEFG-8 monoclonal, MEFG-8 polyclonal, and CD63 antibodies, was performed to measure confirm the identity of the exosomes present in each of the isolates (Figure 3). The protein content in the IP isolate was shown to be the highest, as measured by the MEFE-8 and CD63 antibodies. The TEI showed a significant band against the MEGD-8 monoclonal antibody, but there were no significant differences in other antibodies tested in comparison with SEC. However, for protein content, isolates from TEI showed the highest values, followed by IP and then SEC (Figure 4). Protein content values from the TEI method (mean protein concentration of 0.1914 ± 0.0121) are significantly higher (*p* = 0.012) than the negative control PBS (mean protein concentration is 0.0085 ± 0.0008 mg/mL).

### 3.2. Therapeutic Cargo Properties of Isolated Exosomes

**Figure 5:**
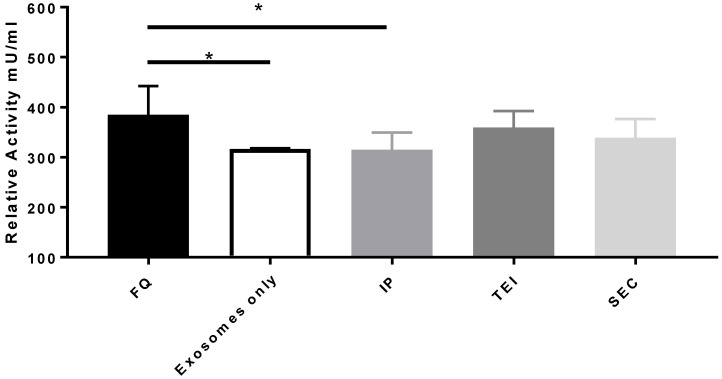
Relative β-galactosidase activity of cells treated with SEC-, IP-, and TEI-isolated EVs loaded with 80 μM quercetin, free 160 μM quercetin, and negative control (PBS). SEC = size exclusion chromatography; IP = immunoprecipitation; TEI = total exosome isolation. All determinations were performed in triplicate; the error bars are the standard deviation. * designates significance at *p* < 0.05.

The cargo properties of the isolated exosomes from different methods were investigated using quercetin-loaded exosome mixtures on pre-cultured and doxorubicin-induced senescent neonatal human dermal fibroblasts. The results show no significant differences in relative β-galactosidase activity between 160 μM of free quercetin compared to 80 μM quercetin-loaded exosomes isolated by SEC (*p* > 0.05) and TEI (*p* > 0.05) (Figure 5), but both are significantly higher than the quercetin loaded with IP and exosomes alone. This indicates that the potential increase (almost doubling) of the intracellular concentration of quercetin loaded in exosomes isolated by SEC and TEI is on par with that of 160 μM free quercetin.

**Figure 6:**
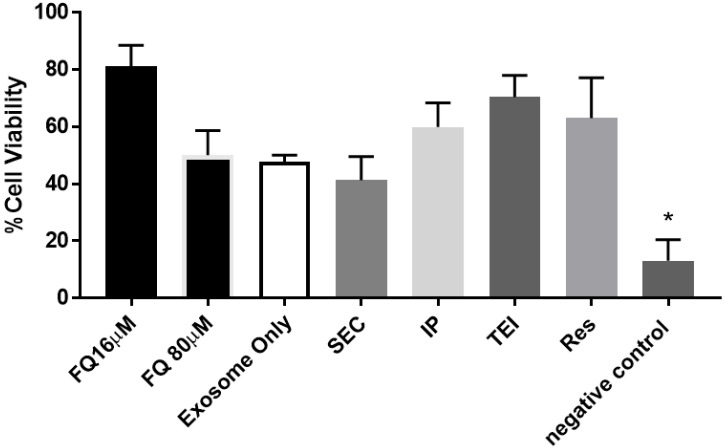
MTT cell viability of cells across treatments with SEC, IP, and TEI loaded with quercetin, free quercetin, and negative controls. SEC = size exclusion chromatography; IP = immunoprecipitation; TEI = total exosome isolation; Res = resveratrol. All determinations were performed in triplicate; the error bars are the standard deviation. * designates significance at *p* < 0.05.

Compared to negative control, all isolated EVs loaded with quercetin significantly increased cell viability (Figure 6). However, quercetin loaded into TEI-isolated exosomes showed a significantly higher cell viability percentage than the other two isolation methods. The viability is similar to that of the 160 μM free quercetin and that of 150 μM resveratrol, a potent antioxidant used as a positive control in this experiment. This finding confirms the concept of exosome’s ability in increasing bioavailability, as well as the reliability of TEI as the most suitable exosome extraction method. Therefore, the TEI extraction method was selected as the most suitable method and, thus, was used for the subsequent in vivo testing.

### 3.3. Organ Histological and Histochemical Analysis

#### 3.3.1. Heart

**Figure 7:**
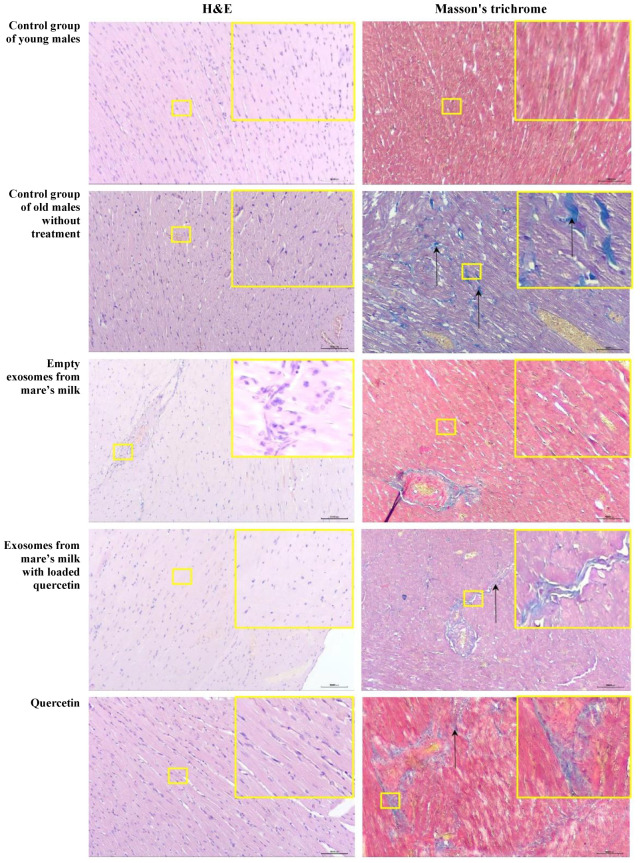
Representative microscopic sections of the left ventricular myocardium. Control group of young males. The histoarchitecture is preserved, and the structure of the heart corresponds to the histological norm. Hematoxylin and eosin, ×100. Lack of collagen fibers in the interstitial space. Masson’s trichrome, ×100. Control group of old males without treatment. Hypertrophy of cardiomyocytes. Hematoxylin and eosin, ×100. Thick, long, moderately crimped collagen fibers of a rich dark blue color (black arrows), randomly located both in the perivascular zones and in the interstitial space. Masson’s trichrome, ×100. Empty exosomes from mare’s milk. Focal perivascular lymphocytic infiltration. Hematoxylin and eosin, ×100. Mild perivascular fibrosis and occasional short light blue collagen fibers in the interstitial space. Masson’s trichrome, ×100. Exosomes from mare’s milk with quercetin included. Histoarchitecture is preserved. Hematoxylin and eosin, ×100. Single short light blue collagen fibers are visible in the interstitial space (black arrows). Masson’s trichrome, ×100. Quercetin. Focal hypertrophy of cardiomyocytes. Hematoxylin and eosin, ×100. Perivascular and interstitial fibrosis: multiple long blue collagen fibers are visible around the vessels and between the cardiomyocytes.

The analysis of the histopathological characteristics of the control untreated group of young male rats showed that the myocardium structure of both the left and right ventricles corresponded to the histological norm. With hematoxylin and eosin staining, the histoarchitecture of the cardiac muscle in all histological sections of all animals was preserved (Table 1). Rows of cardiomyocytes without hypertrophy, significant eosinophilia, or cross striation were identified, with a centrally located basophilic oval vesicular nucleus (Figure 7). With Masson’s trichrome staining, a small number of collagen fibers were observed in the perivascular space, predominantly located in the vessel wall (Table 1). Collagen fibers were mostly absent in the interstitial space. In the individual fields of view of some sections, threadlike thin and short blue collagen fibers were noted (Figure 7).

In the control group of old untreated rats, all animals exhibited bundles of hypertrophied cardiomyocytes, the presence of pyknotic nuclei, and focal hyper-eosinophilia of cardiomyocytes, as well as increased size of cardiomyocyte nuclei and the appearance of multinucleated cardiomyocytes (Figure 7). In some fields of view, individual nuclei were enlarged, from oval to round, hyperchromatic, and had a dense central longitudinal septum (Figure 7), and atherosclerotic changes were identified in the walls of large arteries (Figure 7). In one rat, isolated mononuclear cells were identified in the myocardium (Figure 7).

With Masson’s trichrome staining, collagen fibers in both the left and right ventricular wall were noted in the perivascular zone and interstitial space, arranged in a chaotic and disordered fashion (Table 1). Thick and twisted dark blue collagen fibers in the myocardial interstitial space formed complex anastomoses. A minimal degree of perivascular fibrosis was found in one animal, and a mild degree was found in two of the animals (Figure 7). Mild interstitial fibrosis was observed in all the animals in this group (Figure 7, Table 1).

In the exosome only group, all the rats exhibited cardiomyocyte hypertrophy, i.e., bundles of large longitudinally arranged hypertrophied heart muscle cells with large basophilic reticular nuclei (Figure 7). In addition, two rats showed focal hyper-eosinophilia, and increased size of cardiomyocyte nuclei with the appearance of multinucleated cardiomyocytes was observed, with one animal exhibiting perivascular lymphoplasmacytic infiltration (Figure 7). Mild atherosclerotic changes in large vessels were observed in all cases. A minimal perivascular fibrosis observed in one of the animals and the other two rats showed mild cases of perivascular fibrosis (Figure 7). Interestingly, a mild form of interstitial fibrosis (Figure 7) was observed in all three animals in this group (Table 1).

All of the three rats in the experimental group that received quercetin-loaded exosomes showed increase in the size of cardiomyocyte nuclei with the appearance of multinucleated cardiomyocytes, two exhibited cardiomyocyte hypertrophy and focal hyper-eosinophilia, and one animal displayed perivascular lymphocytic infiltration (Table 1). Focal myocytolysis was also observed: some cardiomyocytes appeared “empty,” i.e., without nuclei and cross striation, and their cytoplasm stained more intensely eosinophilic (Figure 7). Mild myocardial artery atherosclerosis and interstitial fibrosis were observed in all animals, and a minimal degree of perivascular fibrosis was found in one and a mild form in another (Table 1). However, all of the animals in the quercetin-only group exhibited cardiomyocyte hypertrophy, increased size of cardiomyocyte nuclei with the appearance of multinucleated cardiomyocytes, mild myocardial artery atherosclerosis, and mild interstitial fibrosis (Table 1). One of the animals in this group had focal hyper-eosinophilia, and using Masson’s trichrome staining, one animal showed minimal perivascular fibrosis, while the other two showed mild perivascular fibrosis (Table 1).

#### 3.3.2. Kidneys

The comparative histomorphometric characteristics of the kidneys of the untreated control group of young male rats showed the normal histological structures of the renal cortex and medulla (Table 2). Hematoxylin and eosin staining revealed a normal kidney structure with Malpighian bodies consisting of a glomerulus surrounded by Bowman’s capsule. Proximal convoluted tubules with a narrow lumen were lined by pyramidal epithelial cells with acidophilic cytoplasm and basal round vesicular nuclei. Distal convoluted tubules were lined by a relatively larger number of cuboidal epithelial cells with a light acidophilic cytoplasm and central round vesicular nuclei (Table 2).

**Figure 8:**
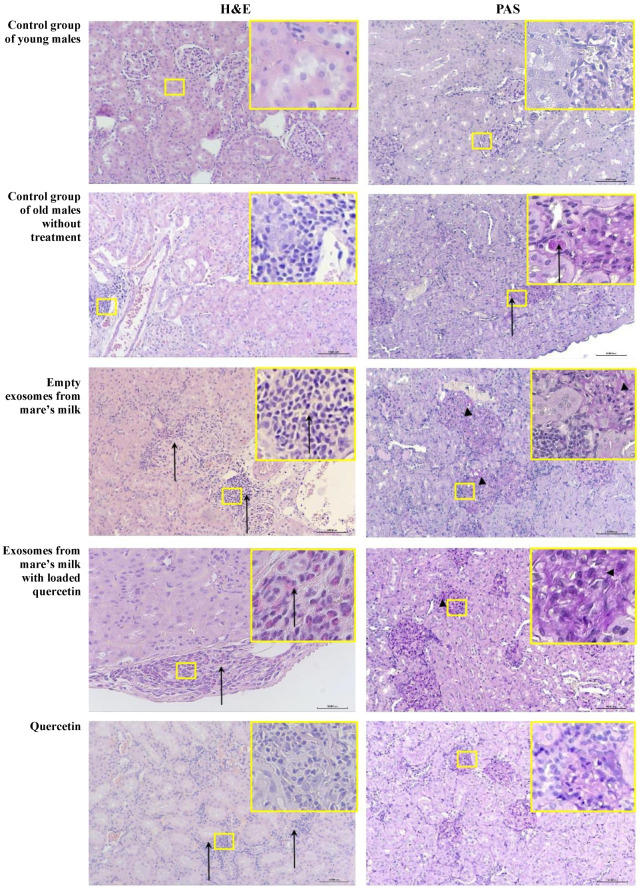
Representative microscopic sections of kidneys. Control group of young males. The structure of the kidney corresponds to the histological norm. Hematoxylin and eosin, ×100.Violet PAS staining of the glomerular mesangial matrix and glomerular basement membranes with minimal staining of the tubular basement membranes. PAS reaction, ×100. Control group of old males without treatment. Focal lymphoplasmacytic infiltration in the interstitium of the kidney. Hematoxylin and eosin, ×100. Formation of protein casts in the renal tubules (black arrows), focal edema and thickening of the basement membranes. PAS reaction, ×100. Empty exosomes from mare’s milk. Focal lymphoplasmacytic infiltration (black arrows) in the interstitium of the renal cortex. Hematoxylin and eosin, ×100. The expansion of the mesangium with an increase in mesangial cells and matrix, hyalinosis of afferent arterioles, thickening and disintegration of the glomerular basement membranes (arrowheads), and focal lymphoplasmacytic infiltration are also present. PAS reaction, ×100. Exosomes from mare’s milk with quercetin included. There is scattered eosinophilic infiltration in the kidney capsule (black arrow). Hematoxylin and eosin, ×100. There is an accumulation of acellular, PAS-positive material, marked thickening of the glomerular basement membranes, edema, and thickening and disintegration of the glomerular basement membranes (arrowheads). PAS reaction, ×100. Quercetin. There is a relatively small number of lymphocytes in the interstitium of the renal cortex, and there is virtually no tissue damage. Hematoxylin and eosin, ×100. Swelling and slight thickening of the glomerular basement membranes. PAS reaction, ×100.

Periodic acid–Schiff (PAS) histochemical staining identified light PAS staining (purple color) of the glomerular mesangial matrix and basal membranes, as well as minimal PAS staining of tubular basal membranes and tubular casts, with no glomerulosclerosis (Figure 8).

In the control group of old untreated male rats, all animals exhibited progressive age-related nephropathy, including regenerative hyperplasia of tubular epithelial cells and the formation of protein casts (Figure 8). Additionally, aging was associated with increased lymphoplasmacytic infiltration in the interstitium of the kidneys. No histopathological signs of tubular epithelial vacuolization were detected in any of the animals in this group.

Sections stained with periodic acid–Schiff (PAS) revealed a PAS-positive reaction (purple–red staining) in the basal membranes and areas of the thickened Bowman’s capsule in all animals, an increase in the number of PAS-stained glomeruli with thickened mesangial matrix and basal membranes, glomerulosclerosis, tubular casts, and PAS-positive thickened tubular basal membranes (Figure 8).

All of the rats in the exosome-only group showed hyperplasia of tubular epitheliocytes, the formation of protein casts, and the presence of lymphoid infiltrates in the renal interstitium, but no tubular vacuolization (Figure 8). Sections stained with periodic acid–Schiff (PAS) revealed thickening and fibrosis of the glomerular basal membranes and glomerulosclerosis in all animals (Table 2). Notably, pronounced eosinophilic infiltration in the kidney capsule was observed in all animals (Table 2), but focal hyalinosis was observed in two animals only (Table 2).

Interestingly, animals in the quercetin-loaded exosomes group showed no tubular epithelial vacuolization, but two animals showed hyperplasia of tubular epitheliocytes, focal cellular infiltration in the renal interstitium, predominantly consisting of lymphatic and plasma cells, thickening and fibrosis of glomerular membranes, and focal hyalinosis (Table 2). The formation of protein casts, glomerulosclerosis, and eosinophilic infiltration were observed in the kidney capsules of all three animals in this group (Table 2).

Animals in the free quercetin-treated group showed no tubular damage, but two animals exhibited hyperplasia of tubular epitheliocytes and the formation of protein casts, and one other animal in the group showed cellular infiltrates predominantly consisting of lymphatic and plasma cells (Table 2). Periodic acid–Schiff (PAS) histochemical staining identified glomerulosclerosis in all animals, focal hyalinosis in one, and, in two animals, edema, thickening and fibrosis of glomerular basal membranes, sclerosis, and hyalinosis were detected (Figure 8).

#### 3.3.3. Liver

**Figure 9:**
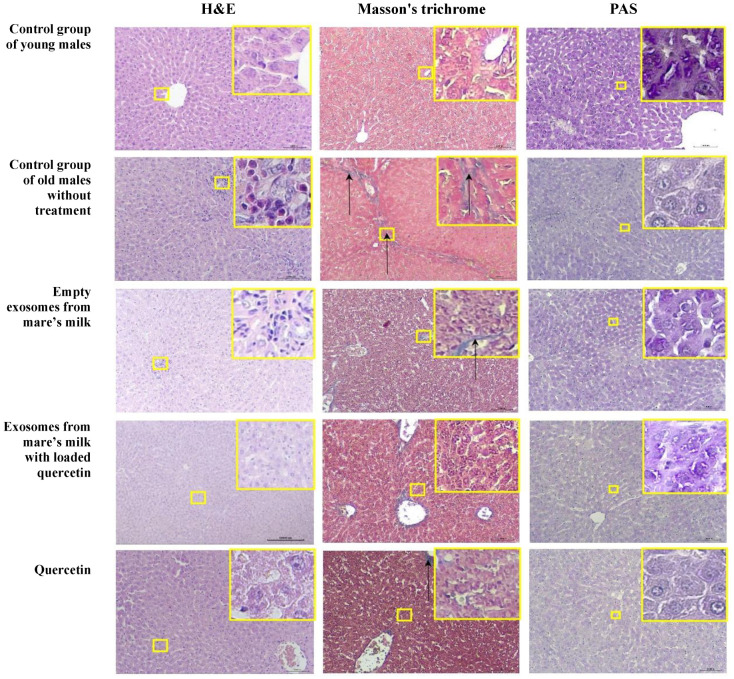
Representative microscopic sections of the liver. Control group of young males. The structure of the liver corresponds to the histological norm. Hematoxylin and eosin, ×100. Blue-stained collagen fibers are visible in the vessel wall but are absent from the extracellular matrix. Masson’s trichrome, ×100. In the cytoplasm of most hepatocytes, multiple glycogen granules are detected in the form of violet–purple particles. PAS reaction, ×100. Control group of old males without treatment. Eosinophilic infiltration of the bile ducts. Hematoxylin and eosin, ×100. Proliferation of collagen fibers in portal tracts with single thick and long collagen fibers (black arrows) forming septa in the extracellular matrix. Masson’s trichrome, ×100. Severe depletion of glycogen in the cytoplasm of hepatocytes. PAS reaction, ×100. Empty exosomes from mare’s milk. Portal tracts with dilated and full-blooded portal veins and focal lymphocytic infiltration. Hematoxylin and eosin, ×100. Collagen fibers (black arrows) are identified in the vessel wall and in the extracellular matrix. Masson’s trichrome, ×100. Moderate amount of glycogen granules in the cytoplasm of hepatocytes. PAS reaction, ×100. Exosomes from mare’s milk with quercetin included. Microvacuolization of the cytoplasm of hepatocytes. Hematoxylin and eosin, ×100. Collagen fibers are found in the vessel wall but are absent from the extracellular matrix. Masson’s trichrome, ×100. In the cytoplasm of hepatocytes, scattered glycogen granules are violet–purple in color. PAS reaction, ×100 Quercetin. Single microvacuoles are detected in the cytoplasm of hepatocytes. Hematoxylin and eosin, ×100. Single filamentous collagen fibers (black arrows) in the extracellular matrix. Masson’s trichrome, ×100. Glycogen granules are detected in the cytoplasm of less than 10% of hepatocytes. PAS reaction, ×100.

The comparative histopathological characteristics of age-related changes in the liver in the studied groups of rats are presented in Table 3. In liver sections of the control group of young male rats, all animals had liver architecture corresponding to the histological norm. With hematoxylin and eosin staining, the liver consisted of numerous hepatic lobules formed by cords of hepatocytes radiating outward from the central vein to the lobule periphery, separated by sinusoids (Table 3). Hepatocytes had a polygonal shape with an eosinophilic granular cytoplasm and vesicular-basophilic nuclei. Masson’s trichrome staining showed collagen fibers in the walls of the portal tract vessels, but these were absent from the extracellular matrix (Table 3). Histochemical staining (PAS) revealed glycogen in the form of violet–purple fragments in the cytoplasm of most hepatocytes in all animals (Figure 9).

Animals in the old untreated group showed evidence of age-related changes with hematoxylin and eosin staining, including hepatocyte vacuolization and inflammatory/immune changes with lymphocyte and plasma cell infiltration (Figure 9 and Table 3). The hepatic sections of all animals in the group were characterized by microvesicular vacuolization of hepatocyte cytoplasm (Table 3). Two out of the three animals in the group displayed some hepatocytes containing nuclei of various shapes and sizes, as well as binucleated hepatocytes in some fields (Figure 9). No signs of central venous congestion were observed in any animal in the group. The portal tracts in all three animals of the group were characterized by dilated and congested portal veins and hyperplasia (cysts) of bile ducts, and an increase in the number of lymphocytes and plasmacytic cells infiltrating between hepatocytes in the portal areas was observed (Table 3). Masson’s trichrome staining showed the proliferation of collagen fibers in the portal tracts with individual thick and long collagen fibers in the extracellular matrix of all animals (Table 3). Histochemical staining (PAS) showed that most hepatocytes were PAS-negative or contained a few scattered glycogen granules of a violet–purple color in less than 10% of hepatocytes in all rats (Figure 9).

However, focal microvesicular vacuolization of hepatocytes and focal lymphocytic infiltration were observed in all animals in the exosome-only group. Cellular pleomorphism occurred, and some of the hepatocytes contained nuclei of variable sizes and shapes, while binucleated hepatocytes and hyperplasia (cysts) in the bile ducts were observed in two of the animals in the group (Table 3). Masson’s trichrome staining in all animals showed perioportal proliferation of collagen fibers (Table 3). Histochemical staining (PAS) showed that most hepatocytes were PAS-negative or contained a small amount of glycogen of a violet–purple color, and in one animal, glycogen was present in less than 61% of hepatocytes (Table 3 and Figure 9).

Interestingly, all animals in the group that was treated with quercetin-loaded exosomes exhibited focal microvesicular vacuolization of hepatocytes, focal lymphocytic infiltration, and hyperplasia or cysts in their bile ducts (Table 3). Two of the animals showed abnormalities in nuclear size and chromatin distribution, as well as binucleated hepatocytes. Masson’s trichrome staining in all animals showed perioportal proliferation of collagen fibers (Table 3). Histochemical staining (PAS) in histological liver sections of two animals revealed glycogen in less than 10% of hepatocytes, and, in one case, in 11–30% of hepatocytes (Table 3). While two of the animals that received free quercetin, exhibited focal microvesicular vacuolization of hepatocytes, binucleated hepatocytes, lymphoplasmacytic infiltration, and hyperplasia (cysts) of bile ducts, all of animals exhibited perioportal fibrosis (Table 3). Glycogen in all animals was detected in less than 10% of hepatocytes (Table 3).

## 4. Discussion

The current study investigated the feasibility of isolating milk-derived exosomes from mare’s milk-derived EVs and tested their suitability as therapeutic carriers. There are other milk and dietary-derived EVs available, but this study focuses on testing the concept of mare’s milk, a widely consumed milk in central Asia. Different exosome isolation methods were used and compared based on the presence of exosomal markers, confirming particle isolation using immunoblotting, particle size via proteomic profiling (Zetasizer, Malvern Panalytical, Malvern, UK), exosome morphology using TEM, and protein concentration via bicinchoninic acid assay (BCA). There are a number of methods available to extract milk-derived exosomes, including centrifugation and an ultracentrifugation density gradient [24], but this study aimed to find a cheap, easy to use and, reliable extraction method. Thus, out of the three tested methods IP, SEC, TEI, the latter proved to be the most suitable methodology capable of isolating high quantity and good quality exosomes.

The suitability of mare’s milk-derived exosomes as therapeutic carriers was tested in terms of an increase in the therapeutic effects compared to a free drug (quercetin only). Quercetin’s vulnerability to changes in pH and poor solubility reduce its bioavailability [25], which appears to be improved by bio-encapsulation, with biomaterials, such as liposomes. Indeed, several studies reported an increase in the therapeutic activity of quercetin-loaded liposomes. For example, de Albuquerque et al., 2023, successfully encapsulated quercetin in lecithin liposome, which improved its availability and biological activity [26]. Similarly, Abd El-Emam et al., 2023, claimed that liposome-loaded quercetin was able to reduce the severity of hepatic damage in rats induced by amoxicillin–clavulanate treatment [27]. In this study, quercetin-loaded exosomes showed significantly higher antioxidant activity compared to quercetin alone in doxorubicin treated cells, demonstrating the potential of exosomes in improving drug bioavailability in vitro. This is due to their ability to easily pass through biological barriers, such as the blood–brain barrier and intestinal and placental barriers [28], which allows them to play an important role in cellular communication and enables them to deliver their mRNA, miRNAs, proteins, and small molecules into other cells [29]. Also, due to the restrictions of the lipid bilayer, exosomes might provide a barrier to protect quercetin from degradation and, hence, overcome its pH instability [25].

This theory was further supported in the animal work, where exosomes isolated from mare’s milk were loaded with quercetin and administered to aging rats. The analysis showed that exosomes loaded with quercetin has more bioavailability than quercetin alone and that they were efficiently absorbed in different organs. This finding is in an agreement with previous reports that showed that the use of milk-derived exosomes as nano-carriers increased drug bioavailability. For example, Munagala et al., 2016, reported that milk-derived exosomes loaded with withaferin A significantly enhanced the tumor reduction potential of the drug [12], while Aqil et al., 2016, showed that the use of exosomes increased the therapeutic response of celastrol against lung cancer [30].

Interestingly, a range of histopathological changes were identified in the heart, liver, and kidney sections of old rats across all groups compared to the group of young rats. These changes included cellular damage (tinctorial and morphological), tissue structure alterations, and cellular infiltration associated with organismal aging. For example, in the heart, the most common age-related lesion observed was fibrosis, with the loss and hypertrophy of cardiomyocytes. The kidneys exhibited focal chronic progressive glomerulonephropathies, including mild sclerosis of the capsule and mesangium of glomeruli. The liver cells displayed widespread variations in the size of hepatocyte nuclei, chromatin distribution within the nucleus, vesicular nuclei, and a basophilic, fine-grained, or vacuolated cytoplasm. The observed damages in the old animals were of an acute, subacute, and chronic nature. While the latter is consistent with age-related damage [31], the acute and subacute are the results of doxorubicin treatment, confirming the success of establishing an aging model using this drug.

However, animals treated with exosomes loaded with quercetin showed significantly less frequent patterns of acute and subacute damage in the myocardium, kidneys, and liver compared to the control group of old males without treatment (*p* < 0.05). These findings were observed in the exosomes loaded with quercetin treatment group, but not with quercetin-only or exosomes-only groups. While this might be used as supporting evidence for the suitability of mare’s milk-derived exosomes as potential drug carriers, it must be interpreted with caution, as exosomes alone were reportedly involved in regulating oxidative stress (OS) [32]. A number of studies indicated that exosomes might not only transport proteins, RNA, and other molecules, but could also participate in OS-related conditions, such as ischemia–reperfusion, atherosclerosis, and cardiac remodeling by reducing reactive oxygen species (ROS) through inhibiting protein synthesis and mRNA degradation [33,34]. For instance, Wang et al., 2019, reported that mir-126 derived from exosomes reduced apoptosis and lessened OS in ischemia and reperfusion injuries [35].

However, the amounts of proteins, mRNA, and RNA found in the exosomes as well as their therapeutic potential depend on the source of the exosomes (e.g., milk) and the isolation methodology used. There were approximately 200 different proteins found in dendritic cell-derived exosomes extracted using a crude preparation [36], and more than 2000 different proteins, including major protein markers, were identified in bovine milk-derived exosomes isolated by centrifugation [37]. The lack of description of the components of the isolated exosomes presents one of the limitations of the current work. Another limitation is that we did not determine the mechanism of action of the exosomes. The latter is currently being investigated in our group. While the protein components of the isolated exosomes were not analyzed, as this is beyond the scope of the current study, we can assume that no proteins or any other component was involved in OS. Thus, it is safe to assume that due to their ability to cross biological barriers, mare’s milk-derived exosomes increased the bioavailability of quercetin, and, hence, they might be used as a reliable form of therapeutic carrier to improve drug delivery.

Nevertheless, the findings confirm the absorption of exosomes by different organs and show an increase in quercetin bioavailability at the target site. However, was the observed effect due to the increased bioavailability of quercetin? Alternatively, did the drug and the exosome achieve it synergistically? Further research is needed to determine whether synergistic effects between exosome and quercetin exist. It would also be interesting to investigate the roles of exosomes isolated from mare’s milk alone, or their components in different pathologies with or without drugs. This will likely shed more light on their biological roles.

## 5. Conclusions

In conclusion, this is the first study to investigate the suitability and efficiency of quercetin-loaded mare’s milk-derived exosomes. The study supports the concept of using exosomes as a potentially reliable form of drug carrier. The results demonstrated that mare’s milk-derived exosomes, can easily be absorbed by different tissues, and that their use as a drug carrier presumably increased the bioavailability of quercetin. However, follow-up studies are needed to identify the components of mare’s milk-derived exosomes, such as proteins, mRNA, and miRNA, as well as to determine their biological roles.

## Figures and Tables

**Table 1:** Morphometric and histopathological assessment of the heart.

Parameters	Scores	Control Group of Young Male Rats (n = 3)	Control Group of Old Male Rats without Treatment (n = 3)	Empty Exosomes from Mare’s Milk (n = 3)	Exosomes from Mare’s Milk Loaded with Quercetin (n = 3)	Quercetin (n = 3)
Hematoxylin and eosin staining						
Cardiomyocyte hypertrophy *	0	100% (3/3)	-	-	33% (1/3)	-
	1	-	100% (3/3)	100% (3/3)	66% (2/3)	100% (3/3)
Focal hyper-eosinophilia *	0	100% (3/3)	-	33% (1/3)	66% (2/3)	66% (2/3)
	1	-	100% (3/3)	66% (2/3)	33% (1/3)	33% (1/3)
Increased size of cardiomyocyte nuclei/appearance of multinucleated cardiomyocytes *	0	100% (3/3)	-	33% (1/3)	66% (2/3)	66% (2/3)
	1	-	100% (3/3)	66% (2/3)	33% (1/3)	33% (1/3)
Cellular infiltrate *	0	100% (3/3)	66% (2/3)	66% (2/3)	66% (2/3)	100% (3/3)
	1	-	33% (1/3)	33% (1/3)	33% (1/3)	-
Masson’s trichrome staining						
Atherosclerosis *	0	100% (3/3)	-	-	-	-
	1	-	100% (3/3)	100% (3/3)	100% (3/3)	100% (3/3)
Perivascular fibrosis **	0	100% (3/3)	-	-	-	-
	1	-	33% (1/3)	33% (1/3)	33% (1/3)	33% (1/3)
	2	-	66% (2/3)	66% (2/3)	66% (2/3)	66% (2/3)
	3	-	-	-	-	-
	4	-	-	-	-	-
Interstitial fibrosis **	0	100% (3/3)	-	-	-	-
	1	-	-	-	-	-
	2	-	100% (3/3)	100% (3/3)	100% (3/3)	100% (3/3)

*—«0»—absent, «1»—present; **—«0»—no change, «1»—minimal changes, «2»—light changes, «3»—moderate changes, and «4»—severe changes.

**Table 2:** Morphometric and histopathological assessment of the kidney.

Parameters	Scores	Control Group of Young Male Rats (n = 3)	Control Group of old Male Rats without Treatment (n = 3)	Empty Exosomes from Mare’s Milk (n = 3)	Exosomes from Mare’s Milk Loaded with Quercetin (n = 3)	Quercetin (n = 3)
Hematoxylin and eosin staining						
Vacuolization of the epithelium	0	100% (3/3)	100% (3/3)	100% (3/3)	100% (3/3)	100% (3/3)
	1	-	-	-	-	-
Hyperplasia of tubular epithelial cells	0	100% (3/3)	-	-	33% (1/3)	33% (1/3)
	1	-	100% (3/3)	100% (3/3)	66% (2/3)	66% (2/3)
Formation of protein casts in tubules	0	100% (3/3)	-	-	-	33% (1/3)
	1	-	100% (3/3)	100% (3/3)	100% (3/3)	66% (2/3)
Cellular infiltrate in the interstitium	0	100% (3/3)	-	-	33% (1/3)	66% (2/3)
	1	-	100% (3/3)	100% (3/3)	66% (2/3)	33% (1/3)
Masson’s trichrome staining						
Thickening and fiberization of basement membranes	0	100% (3/3)	-	-	33% (1/3)	33% (1/3)
	1	-	100% (3/3)	100% (3/3)	66% (2/3)	66% (2/3)
Hyaline glomerulopathy	0	100% (3/3)	33% (1/3)	33% (1/3)	33% (1/3)	66% (2/3)
	1	-	66% (2/3)	66% (2/3)	66% (2/3)	33% (1/3)
Glomerulosclerosis	0	100% (3/3)	-	-	-	-
	1	-	100% (3/3)	100% (3/3)	100% (3/3)	100% (3/3)

«0»—absence, and «1»—presence.

**Table 3:** Morphometric and histopathological assessment of the liver.

Parameters	Scores	Control Group of Young Male Rats (n = 3)	Control Group of Old Male Rats without Treatment (n = 3)	Empty Exosomes from Mare’s Milk (n = 3)	Exosomes from Mare’s Milk Loaded with Quercetin (n = 3)	Quercetin (n = 3)
Hematoxylin and eosin staining						
Vacuolization of hepatocytes *	0	100% (3/3)	-	-	-	33% (1/3)
	1	-	100% (3/3)	100% (3/3)	100% (3/3)	66% (2/3)
Karyocytomegaly and/or multinucleated hepatocytes *	0	100% (3/3)	33% (1/3)	33% (1/3)	33% (1/3)	33% (1/3)
	1	-	66% (2/3)	66% (2/3)	66% (2/3)	66% (2/3)
Central venous stasis *	0		-	-	-	-
	1		100% (3/3)	100% (3/3)	100% (3/3)	100% (3/3)
Cellular infiltrate *	0	100% (3/3)	-	-	-	33% (1/3)
	1	-	100% (3/3)	100% (3/3)	100% (3/3)	66% (2/3)
Masson’s trichrome staining						
Bile duct hyperplasia/cysts *	0	100% (3/3)	-	33% (1/3)	-	33% (1/3)
	1	-	100% (3/3)	66% (2/3)	100% (3/3)	66% (2/3)
Periportal fibrosis *	0	100% (3/3)	-	-	-	-
	1	-	100% (3/3)	100% (3/3)	100% (3/3)	100% (3/3)
Glycogen accumulation **	0	-	100% (3/3)	66% (2/3)	66% (2/3)	100% (3/3)
	1	-	-	-	33% (1/3)	-
	2	-	-	33% (1/3)	-	-
	3	100% (3/3)	-	-	-	-

*—«0»—absent, «1»—present; **—«0»-less than 10%, «1»—11–30%, «2»—31–60%, and «3»—more than 61%.

## Data Availability

The data that support the findings of this study are available from the corresponding author upon reasonable request.

## Supplementary Materials

The following supporting information can be downloaded at: https://www.mdpi.com/article/10.3390/biom14101247/s1, Figure S1: Western blots images.
