# Supplementary figures and images for: Investigating the Suitability of Mare’s Milk-Derived Exosomes as Potential Drug Carriers
Source: Biomolecules. 2024 Oct 1;14(10):1247. doi: 10.3390/biom14101247 (PMC11506534; doi:10.3390/biom14101247)

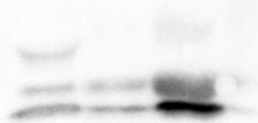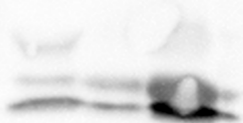

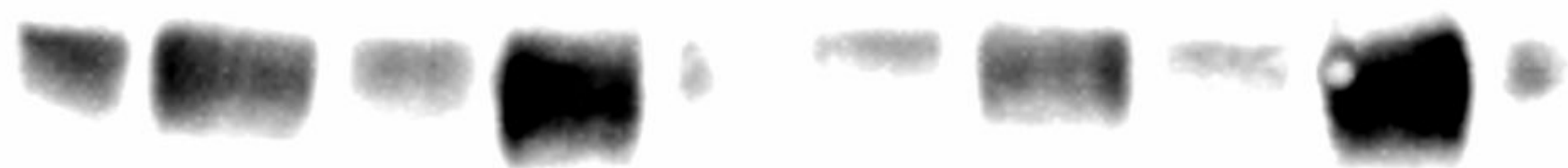

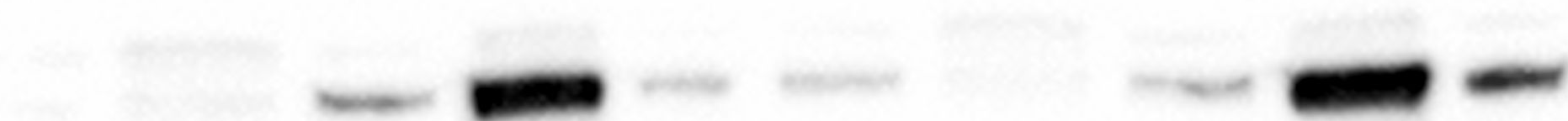

Supplement: Supplementary file 1 [file biomolecules-14-01247-s001.zip › biomolecules-3197800-supplementary.pdf]
